# Podcast‐induced reflex seizures: A unique trigger

**DOI:** 10.1002/epd2.70254

**Published:** 2026-05-04

**Authors:** David W. Cao, Roberto Gonzalez, Irfan S. Sheikh, Melissa Huynh Mabry

**Affiliations:** ^1^ Division of Epilepsy, Department of Neurology University of Texas Southwestern Medical Center Dallas Texas USA; ^2^ Peter O’Donnell Jr. Brain Institute, University of Texas Southwestern Medical Center Dallas Texas USA

**Keywords:** etiology: structural, localization: medial temporal lobe, amygdala, phenomenology: aura, focal impaired consciousness, tonic clonic seizures, syndrome: reflex epilepsy

Reflex seizures (RS) are epileptic events that are consistently triggered by identifiable stimuli. Here, we present a case of reflex seizures that are triggered by Christian sermon podcasts. Informed consent was obtained from the patient.

This is a 56‐year‐old woman with a history of depression, hypothyroidism, and migraines who has been followed in our clinic for drug‐resistant epilepsy for the past 24 years. She has three main seizure semiologies: an aura of burning smell with deja vu, focal impaired consciousness (FIC) seizures that can rarely progress to focal to bilateral tonic clonic seizures (FTBTC). The patient reported that her seizures were consistently triggered by male narrating voices and in particular Christian sermons conducted with a male pastor.

Of note, she also has comorbid psychogenic non‐epileptic seizures, which are characterized similar to her epileptic seizures but with preserved consciousness during the event.

She had no family history of epilepsy. A focused neurologic examination revealed no abnormalities.

She was admitted to our epilepsy monitoring unit (EMU) in 2025 and four seizures (3 FIC and 1 FTBTC) were captured; all were triggered by male Christian sermon with localization to the right temporal lobe. Her captured seizure semiology consisted of initially listening to male Christian sermons. Her FIC seizures consisted of oroalimentary automatisms, bilateral hand trembling, and a post‐ictal nose wipe using her right hand. Her FIC seizure could progress with a FTBTC seizure with a leftward versive head turn with left gaze deviation. She also had interictal findings of right temporal sharp waves, lateralized periodic discharges, and focal slowing. She was discharged from the EMU with an increase in her anti‐seizure medications to include lacosamide 200 mg twice a day and brivaracetam 50 mg twice a day (Video 1).

**Video 1:** Video electroencephalogram of a reflex seizure episode triggered by listening to a Christian sermon. Video content can be viewed at https://onlinelibrary.wiley.com/doi/10.1002/epd2.70254.

Her workup to this point has consisted of multiple brain MRIs since 2009, which were notable for hyperintense T2/FLAIR signal involving the right medial temporal lobe and amygdala, which was thought to be secondary to post‐ictal changes. She has also received PET brain imaging with subtle right temporal hypometabolism and a magnetoencephalography study with a tight cluster of epileptiform discharges with stable orientation in the right middle and superior temporal gyrus. Her neuropsychological testing was broadly intact without lateralizing or localizing deficits, despite right temporal localization on other modalities (possibly reflecting compensatory reorganization over her 24‐year epilepsy course). Her genetic testing revealed three variants of unknown significance in the GATAD2B, GLRA1, and GRIN2D, which were felt to be non‐contributory.

The stimuli and triggers for RS are widely heterogeneous. The stimuli may be external (triggered by sensory stimuli) or internal (triggered by emotions or cognitive processes), and the epileptic activity may be generalized or focal (with or without secondary generalization).^1^ The most common stimuli reported in RS are visual stimuli (flashing lights), making up 75–80% of RS.^1^, ^2^ RS induced by complex emotional and auditory stimuli, while significantly less common, is becoming increasingly recognized.^1^, ^3^, ^4^

Our case highlights a rare form of reflex seizures precipitated by emotionally significant auditory and visual stimuli, specifically Christian audio podcasts. While musicogenic seizures (i.e., RS triggered by music) have been described in the literature, there have been few reports of spoken auditory stimuli as seizure triggers.^1^, ^5^ More specifically, to our knowledge, there have been no reports of Christian audio podcasts, or any podcasts, as seizure triggers. This case suggests that emotionally charged speech, even in the absence of musical motifs, may be sufficient to generate seizures within the temporal lobes. Previous studies have shown that emotionally charged music is capable of provoking RS with fMRI/EEG demonstrating activation fronto‐temporo‐occipital networks, and it has been theorized that musicogenic seizures may be a result of hyperactivation of the mesolimbic system and the associated auditory‐limbic networks.^6^, ^7^ In a patient where certain topics may carry additional emotional significance, the auditory stimulus of a spoken podcast may similarly activate these networks and lower the seizure threshold.

Management of reflex seizures of this variety primarily involves avoidance of the auditory stimuli. If unable to do so, or in patients with another epilepsy syndrome, antiseizure medications may be used as seen with our patient, although she is currently under evaluation for surgical treatment options.^1^ This case adds to the limited literature on RS triggered by non‐musical spoken stimuli.

## AUTHOR CONTRIBUTIONS

**David W. Cao:** Writing—Original Draft, Visualization **Roberto Gonzalez:** Writing—Original Draft, Visualization **Irfan S Sheikh:** Supervision, Writing—Review & Editing **Melissa Huynh Mabry:** Conceptualization, Project Administration, Writing—Review & Editing.

## FUNDING INFORMATION

The authors received no financial support for the research, authorship, and/or publication of this article.

## CONFLICT OF INTEREST STATEMENT

Sheikh IS an associated editor for Epileptic Disorders Journal and is the assistant program leader of the Epileptic Disorders Internship Program. The authors have no other conflicts of interest to note.

## PATIENT CONSENT

Patient informed consent was obtained.

**Test yourself**

1. Which of the following best characterizes reflex seizures (RS)?
  - Seizures that occur exclusively in response to emotional stress
  - Seizures that are consistently triggered by identifiable stimuli
  - Seizures that only occur in patients with generalized epilepsy syndromes
  - Seizures that are provoked by metabolic derangements
  - Seizures that occur exclusively in childhood
2. Reflex seizures triggered by emotionally salient auditory stimuli (e.g., music or spoken speech) are hypothesized to involve which of the following neural networks?
  - Primary somatosensory cortex and dorsal column pathways
  - Cerebellothalamic motor circuits
  - Mesolimbic and auditory‐limbic networks
  - Brainstem vestibular nuclei
  - Occipital‐striatal visual processing pathways
3. Which of the following statements regarding reflex seizures is most accurate?
  - Reflex seizures are most commonly triggered by complex emotional auditory stimuli.
  - Reflex seizures are always generalized in onset.
  - Visual stimuli account for the majority of reported reflex seizure triggers.
  - Reflex seizures require an underlying genetic epilepsy syndrome.
  - Avoidance of triggers is ineffective in preventing recurrence.

*Answers may be found in the* *supporting information*.

## Supporting information


Data S1:

Data S2:

## Data Availability

Data supporting this study is included within the article and/or supporting materials. Any further requests for anonymized patient data regarding this case report can be made to the corresponding author as appropriate. These data are not publicly available due to privacy or ethical restrictions.
