# Supplementary material for: Podcast‐induced reflex seizures: A unique trigger
Source: Epileptic Disord. 2026 May 4;28(4):1316–8. doi: 10.1002/epd2.70254 (PMC13499241; doi:10.1002/epd2.70254)
Supplement: Supplementary file 1 [file EPD2-28-1316-s001.docx]

Question 1

Which of the following best characterizes reflex seizures (RS)?

A. Seizures that occur exclusively in response to emotional stress
B. Seizures that are consistently triggered by identifiable stimuli
C. Seizures that only occur in patients with generalized epilepsy syndromes
D. Seizures that are provoked by metabolic derangements
E. Seizures that occur exclusively in childhood

Question 2

Reflex seizures triggered by emotionally salient auditory stimuli (e.g., music or spoken speech) are hypothesized to involve which of the following neural networks?

A. Primary somatosensory cortex and dorsal column pathways
B. Cerebellothalamic motor circuits
C. Mesolimbic and auditory-limbic networks
D. Brainstem vestibular nuclei
E. Occipital-striatal visual processing pathways

Question 3

Which of the following statements regarding reflex seizures is most accurate?

A. Reflex seizures are most commonly triggered by complex emotional auditory stimuli.
B. Reflex seizures are always generalized in onset.
C. Visual stimuli account for the majority of reported reflex seizure triggers.
D. Reflex seizures require an underlying genetic epilepsy syndrome.
E. Avoidance of triggers is ineffective in preventing recurrence.

Answer Key:

Question 1

Correct Answer: B

Explanation: Reflex seizures are epileptic events that are consistently and reproducibly triggered by specific identifiable stimuli. These stimuli may be external (e.g., visual or auditory) or internal (e.g., emotional or cognitive processes), and seizure onset may be focal or generalized.

Question 2

Correct Answer: C

Explanation: Emotionally meaningful auditory stimuli are thought to activate mesolimbic structures and interconnected auditory-limbic networks. In susceptible individuals, hyperactivation of these temporal lobe–associated circuits may lower seizure threshold and precipitate reflex seizures.

Question 3

Correct Answer: C

Explanation: Visual stimuli, particularly flashing lights, account for approximately 75–80% of reported reflex seizure triggers. Reflex seizures may be focal or generalized, and management often includes trigger avoidance in addition to antiseizure medications when appropriate.
